# Role of CMR Mapping Techniques in Cardiac Hypertrophic Phenotype

**DOI:** 10.3390/diagnostics10100770

**Published:** 2020-09-29

**Authors:** Andrea Baggiano, Alberico Del Torto, Marco Guglielmo, Giuseppe Muscogiuri, Laura Fusini, Mario Babbaro, Ada Collevecchio, Rocco Mollace, Stefano Scafuri, Saima Mushtaq, Edoardo Conte, Andrea Daniele Annoni, Alberto Formenti, Maria Elisabetta Mancini, Giulia Mostardini, Daniele Andreini, Andrea Igoren Guaricci, Mauro Pepi, Marianna Fontana, Gianluca Pontone

**Affiliations:** 1 Cardiovascular Imaging Department, Centro Cardiologico Monzino IRCCS, 20138 Milan, Italy; andrea.baggiano@ccfm.it (A.B.); alberico.deltorto@ccfm.it (A.D.T.); marco.guglielmo@ccfm.it (M.G.); giuseppe.muscogiuri@ccfm.it (G.M.); laura.fusini@ccfm.it (L.F.); saima.mushtaq@ccfm.it (S.M.); edoardo.conte@ccfm.it (E.C.); andrea.annoni@ccfm.it (A.D.A.); alberto.formenti@ccfm.it (A.F.); maria.mancini@ccfm.it (M.E.M.); giulia.mostardini@ccfm.it (G.M.); daniele.andreini@ccfm.it (D.A.); mauro.pepi@ccfm.it (M.P.); 2 Dipartimento di Medicina Clinica e Molecolare, Facoltà di Medicina e Psicologia, Università degli Studi di Roma “Sapienza”—Azienda Ospedaliera Sant’Andrea, 00189 Rome, Italy; babbaromario@gmail.com; 3 Department of Cardiac, Thoracic, Vascular Sciences and Public Health, University of Padua, 35128 Padua, Italy; ada.collevecchio@gmail.com; 4 Department of Cardiology, University of Rome Tor Vergata, 00133 Rome, Italy; rocco.mollace@gmail.com; 5 Cardiothoracovascular Department, University of Florence, Azienda Ospedaliero-Universitaria Careggi, 50139 Florence, Italy; scafuristefano90@gmail.com; 6 Cardiovascular Section, Department of Clinical Sciences and Community Health, University of Milan, 20122 Milan, Italy; 7 Institute of Cardiovascular Disease, Department of Emergency and Organ Transplantation, University Hospital Policlinico of Bari, 70124 Bari, Italy; andrea.guaricci@gmail.com; 8 National Amyloidosis Centre, Division of Medicine, University College London, Royal Free Hospital, London NW3 2PF, UK; m.fontana@ucl.ac.uk

**Keywords:** cardiovascular magnetic resonance, non-ischemic cardiomyopathies, hypertrophic phenotype, T1 mapping, T2 mapping, ECV mapping

## Abstract

Non-ischemic cardiomyopathies represent a heterogeneous group of myocardial diseases potentially leading to heart failure, life-threatening arrhythmias, and eventually death. Myocardial dysfunction is associated with different underlying pathological processes, ultimately inducing changes in morphological appearance. Thus, classification based on presenting morphological phenotypes has been proposed, i.e., dilated, hypertrophic, restrictive, and right ventricular cardiomyopathies. In light of the key diagnostic and prognostic role of morphological and functional features, cardiovascular imaging has emerged as key element in the clinical workflow of suspected cardiomyopathies, and above all, cardiovascular magnetic resonance (CMR) represents the ideal technique to be used: thanks to its physical principles, besides optimal spatial and temporal resolutions, incomparable contrast resolution allows to assess myocardial tissue abnormalities in detail. Traditionally, weighted images and late enhancement images after gadolinium-based contrast agent administration have been used to perform tissue characterization, but in the last decade quantitative assessment of pre-contrast longitudinal relaxation time (native T1), post-contrast longitudinal relaxation time (post-contrast T1) and transversal relaxation time (T2), all displayed with dedicated pixel-wise color-coded maps (mapping), has contributed to give precious knowledge insight, with positive influence of diagnostic accuracy and prognosis assessment, mostly in the setting of the hypertrophic phenotype. This review aims to describe the available evidence of the role of mapping techniques in the assessment of hypertrophic phenotype, and to suggest their integration in the routine CMR evaluation of newly diagnosed cardiomyopathies with increased wall thickness.

## 1. Introduction

Cardiovascular diseases represent the major causes of mortality and morbidity both for men and women in the European Countries [1]. Undeniably, ischemic heart disease accounts for nearly the half of the cases, while the remaining percentage is split between stroke and non-ischemic heart diseases1. Among the latter, several inherited and acquired processes not primarily related to coronary atherosclerosis have been recognized as cause of myocardial dysfunction, potentially leading to heart decompensation, life-threatening arrhythmias, and eventually death [2]. From a pathophysiological perspective, myocardial dysfunction can be related to disproportionate myocytes hypertrophy not induced by abnormal loading conditions [3], extracellular space infiltration of abnormal protein fibrils [4,5,6], intracellular glycosphingolipid accumulation [7], intracellular iron deposition [8], focal or diffuse fibrotic [9], or fibrofatty [10] replacement.

Such tissue abnormalities lead to changes in morphological appearance, thus classifications based on morphological features (i.e., dilated, hypertrophic, restrictive, and right ventricular cardiomyopathies) have been proposed [11,12,13]. Lately the key role of cardiovascular imaging in managing these cardiomyopathies has been highlighted, not only from a diagnostic perspective, but also for prognostic purpose [14,15,16,17,18,19].

Above different imaging modalities, cardiac magnetic resonance (CMR) emerged as the ideal technique to assess myocardial diseases, not only because it provides precise and highly reproducible measurements (e.g., wall thickness, chamber size, ejection fraction, valvular regurgitation, and stenosis), but also because, due to inherent physical principles, it allows to generate contrast between different tissues. According to different longitudinal and transverse relaxation times, properties specific for each tissue, it is possible to set pulse sequence parameters, such as repetition time (TR), echo time (TE) and flip angle, in order to magnify the signal from a particular tissue [20]. Thanks to these features, it is possible to describe the presence of tissue abnormalities such as edema or fatty infiltration [20]. Moreover, the administration of gadolinium-based contrast agent, a highly paramagnetic substance, carried within the blood pool into the extracellular space of the myocardial muscle, allow to differentiate between normal myocardium and abnormal/damaged tissue at delayed images acquisition [21].

Recently, technical advances in magnetic resonance imaging have made it possible to quantify longitudinal relaxation time (T1) in the heart and to generate pixel-wise color-encoded maps, in which the pixel values represent the T1 magnitude in each voxel, depicting even relatively small variations within the myocardium, allowing detailed tissue pathology description [22]. Indeed, this technique, called “native T1 mapping” as obtained without paramagnetic contrast agent administration, is sensitive to different types of myocardial tissue damages, such as oedema, iron overload, amyloid and fat infiltration, myocardial scars due to ischemic and non-ischemic damages [23,24]. For these reasons, T1 mapping is becoming a precious tool for a complete myocardial evaluation when assessing a patient with suspected or known myocardial disease. Interestingly, tissue abnormalities quantification through T1 mapping allows to track myocardial changes in the same patient over time, appealing aspect for using this tool in pharmacological and treatment trials.

Moreover, it is possible to exploit the longitudinal relaxation time shortening effect induced by the administration of extracellular paramagnetic contrast agent to obtain a “post-contrast” T1 map; once data from pre-contrast and post-contrast maps are co-registered, along with the knowledge of blood hematocrit, a direct measure of the extracellular volume (ECV) is obtained, representing a quantification of the relative expansion of the cardiac extracellular matrix [23].

In addition, considering the several disadvantages of T2-weighted (T2W) spin echo sequences, historically used to detect tissue oedema, like signal intensity variability caused by phased array coils, high signal from slow moving blood into ventricular chambers, motion artefacts and, mostly, the subjective nature of T2W image interpretation, also the transversal relaxation time (T2) has been assessed with a quantitative approach, and T2 mapping resulted in an excellent method for depicting myocardial segments affected by an increase in water content, as during acute myocardial injuries [25,26].

The result of this “multiparametric mapping approach” is the creation of a sort of non-invasive myocardial biopsy, intended to be a crucial step in the diagnostic workflow of cardiac diseases.

As expected from such a promising tool, mapping techniques have been applied to many cardiac diseases. The present review, although by no means exhaustive, presents an overview of the mapping techniques application in the setting of myocardial disease characterized by a left ventricle (LV) hypertrophic phenotype not induced by loading conditions, suggesting the integration of these tools in the routine CMR evaluation of newly diagnosed cardiomyopathies with increased wall thickness.

## 2. Brief Technical Aspects

### 2.1. T1 Mapping and ECV Mapping

The measurement of the longitudinal relaxation time, called T1, as a marker of changes in tissue structure and composition, coupled with the technical chance of creating pixel-wise parametric maps, registering computed T1 values to the underlying source images, made it possible to deliver to physicians a detailed picture of cardiac muscle composition [22].

Briefly, the general principle for T1 mapping is to acquire multiple images with different T1 weightings and then to fit the signal intensities of the images to a dedicated equation [27]. For obtaining T1 measurements, the equilibrium magnetization is either inverted or nulled with RF pulses, and subsequently T1-weighted images are acquired at different times after the inversion or saturation pulse. Finally, T1 times can be determined for regions of interest, myocardial segments, or, preferably, at each pixel location in order to form a T1 map, where pixel intensities correspond to fitted T1 values [28].

Many sequences have been developed to obtain T1 mapping, and several pulses schemes have been tested [29]. Above all, two of them have been adopted by the majority of CMR physicians worldwide, MOLLI and ShMOLLI: MOLLI (MOdified Look-Locker Inversion recovery sequence) is composed by single-shot images acquired intermittently in diastole during 3 to 5 heartbeats after the inversion pulse, resulting in images spaced by the RR-interval along the T1 recovery curve, with the most popular MOLLI variant, the 5(3)3 one, requiring 11 heartbeats to complete the T1 mapping image; ShMOLLI (“shortened” MOLLI) is characterized by a 5(1)1(1)1 acquisition scheme (3 Look-Locker cycles over 9 heartbeats), resulting in modest heart rate dependence and requiring a shorter breath-hold [29,30].

Combining pre-contrast and post-contrast T1 data of the myocardium, focusing on the change of the T1 relaxation rate (i.e., R = 1/T1) of the blood between pre- and post-contrast imaging, and using the blood haematocrit (Hct) to derive plasma T1 (a reference for the T1 changes in tissue), the estimation of the extracellular volume (ECV) can be obtained with the following formula: (1 − Hct) × (ΔRm/ΔRb), where ΔRm represents the change in myocardial T1 relaxation rate after contrast administration (ΔRm = R1post − R1pre), and ΔRb represents the same variation in the blood [31]. With this approach, it is possible to obtain an estimation of the entity of an interstitial space (the space within the myocardium once the intracellular and intravascular compartments are removed). Changes in ECV are usually induced by changes in the interstitial volume fraction, acting as surrogate marker of interstitial remodeling and interstitial fibrosis, so its use has been proved to be very useful in understanding many myocardial diseases.

### 2.2. T2 Mapping

Compared to T1 mapping, T2 mapping technique is more standardized. Methods for T2 mapping include T2-prepared steady-state free precession (SSFP) [32], multiecho spin echo (MESE) [33], and a hybrid of MESE and either turbo spin echo [34] or gradient spin echo (GraSE) [35]. The most widely used method is T2-prepared SSFP: to generate a T2 map, a SSFP sequence is used to produce three single-shot T2-weighted images, each of them with a different T2 preparation time (TE_T2P_), usually 0 ms, 25 ms, and 55 ms [36]. The images, acquired in the same cycle phase, in a single apnoea, and in successive heart beats, are obtained every 2 to 4 RR intervals, according to heart rate, in order to achieve sufficient T1 recovery [37], and then nonrigid movement-correction algorithm is applied to correct residual heart and respiratory movement. Once the images are processed adjusting the T2 recovery curve with each pixel, a T2 mapping image is generated.

**Figure 1:**
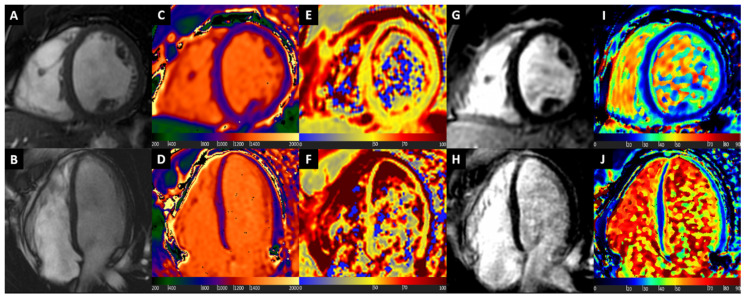
Normal heart. Basal short axis view (Box **A**) and 4-chamber long axis view (Box **B**) balanced steady state free precession images showing normal biventricular size and wall thickness. Normal T1 values at native T1 mapping images (Boxes **C** and **D**). Normal T2 values at T2 mapping (Boxes **E** and **F**), excluding the presence of oedema. Late gadolinium enhancement (LGE) images show uniform myocardial suppression (Boxes **G** and **H**). Normal extracellular volume (ECV) values at ECV mapping (Boxes **I** and **J**).

A representative case of a patient with normal morphological ventricular appearance, along with normal non-contrast tissue characterization with T1 and T2 mapping, and post-contrast assessment with late gadolinium enhancement (LGE) and ECV mapping is displayed in Figure 1.

## 3. Hypertrophic Cardiomyopathy

Hypertrophic cardiomyopathy (HCM) is a genetically determined heart muscle disease characterized by increased left ventricular wall thickness that is not solely explained by abnormal loading conditions, and is caused in 60% of cases by mutations in cardiac sarcomere protein genes [14], with an estimated prevalence of at least 0.5% in the general population [38]. While many patients with HCM are asymptomatic, some patients may present with heart failure symptoms, angina, palpitations, syncope, and sudden cardiac death. As HCM is defined by a wall thickness ≥15 mm in at least one left ventricular myocardial segment, imaging methods are central to the diagnosis. Compared to echocardiography, CMR can measure LV wall thickness more precisely, especially in segments less reliably visualized by echocardiography such as the apical segments [39,40]. After gadolinium-based contrast injection, LGE is often found in hypertrophic segments, in a non-ischemic pattern (usually patchy/mid-wall enhancement) [41]. LGE in hypertrophic segments has been correlated to focal gross myocardial structure abnormalities, with interstitium expansion due to fibrosis [42], and LGE extension of ≥15% of LV mass has been associated with a 2-fold increase in sudden cardiac death risk, even in patients without additional risk markers [43].

Assessment of HCM patients with mapping sequences allowed physicians to go beyond focal tissue abnormalities. Dass et al. first showed that native T1 was diffusely mildly increased in HCM patients compared to healthy controls, regardless the presence of LGE and hypertrophy; however, with the most relevant increase in hypertrophic segments and in segments with LGE [44]. Another study from Kato et al. proved that more than 30% of myocardial LGE negative segments showed increased native T1 values, although confirming a significant correlation between LV wall thickness and native T1 [45]. Furthermore, Huang et al. showed that both T1 and T2 values are increased in HCM patients, despite normal wall thickness and preserved contraction function, suggesting that tissue remodeling may precede morphological and functional remodeling in HCM patients, thus highlighting the additional role of native T1 and T2 mapping for HCM diagnosis at an early stage [46]. In support of the concept of T1 mapping capability to look “deep” into the myocardium, a recent study by Wang et al. showed that newly-developed radiomic analysis of native T1 values could differentiate between HCM induced by mutation of MYH7 (β-myosin heavy chain) or MYBPC3 (β-myosin-binding protein C), the most commonly affected genes, with good discrimination [47]. Anyway, it must be underlined how entity and distribution of increase of native T1 mapping does not always allow to differentiate between HCM and other myocardial disease, suggesting that mapping techniques should be merged with clinical, morphological and LGE information to be correctly interpreted [48,49].

While Huan et al. showed that T2 mapping could play a role in supporting the early diagnosis of HCM [46], Amano et al. demonstrated that the presence of increased T2 values in HCM patients was associated with an increase in troponin T and brain natriuretic peptide levels (BNP), underlining the potential role of T2 information in recognizing more aggressive forms characterized by significant myocardial injury [50].

Considering the myocardial structure abnormalities in HCM, also post-contrast T1 values changes, influenced by many factors including fibrosis, have been investigated for a potential clinical role [23]. In a recent study, McLellan et al. showed that in 100 patients with HCM, followed-up for 40 months, post-contrast T1 values were significantly lower in patients with non-sustained ventricular tachycardia (NSVT) and sudden cardiac death (SCD), being predictor of NSVT at multivariate analysis [51], and therefore having been proposed as a possible tool for risk stratification.

Moreover, ECV, reflecting purely interstitium compartment and site of fibrosis expansion in various myocardial disease, has been assessed in HCM [23]. As expected, Sado et al. showed that ECV is higher in HCM compared to healthy controls and that its values are correlated with the percentage of LGE [52]. Interestingly, Swoboda et al. showed that ECV values are negatively correlated with maximum wall thickness in competitive athletes, often characterized by mildly hypertrophied myocardial walls, while there is a positive correlation in HCM patients [53]. Therefore, based on this divergent finding, ECV has been suggested to be used to distinguish HCM, in which hypertrophy is mediated by cellular disarray and extracellular matrix expansion, and athletic remodeling, in which the increase in LV mass in healthy myocardium is mediated by cellular hypertrophy [53]. Avanesov et al. studied the ability of ECV to predict prognosis in HCM patients, and then compared it to other CMR and conventional risk markers. In 73 subjects affected by HCM followed-up for 5 years, ECV was proved to be a better predictor of SCD than LGE and performed similarly to HCM Risk-SCD score and better than LGE and post-contrast T1 mapping in predicting syncope or NSVT [54]. Moreover, an incremental value of combined analysis of HCM Risk-SCD score and ECV for prediction of syncope and NSVT was shown [54], underlining a possible role of routine ECV analysis in HCM patients to improve the patient selection for implantable cardioverter-defibrillator (ICD) implantation.

According to the evidence available, even if current international guidelines do not clearly recommend advanced CMR tissue characterization but solely pronounce on LGE analysis, a comprehensive CMR scan with mapping sequences and LGE should be performed in every patient with suspected HCM.

**Figure 2:**
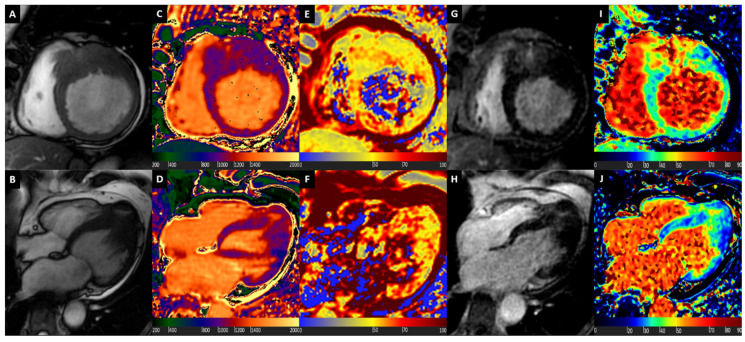
Hypertrophic Cardiomyopathy. Basal short axis view (Box **A**) and 4-chamber long axis view (Box **B**) balanced steady state free precession images showing asymmetric myocardial hypertrophy. Diffuse mild increase of T1 values at native T1 mapping images, with highest values at hypertrophied segments (Boxes **C** and **D**). Myocardial oedema detected by T2 mapping (Boxes **E** and **F**), mostly at septal level. LGE images show mid-wall patchy enhancement at hypertrophic segments (Boxes **G** and **H**). Diffuse mild increase of ECV, with highest values at hypertrophied segments (Boxes **I** and **J**).

A representative case of patient with HCM is illustrated in Figure 2.

## 4. Cardiac Amyloidosis

Cardiac amyloidosis (CA) is a disease characterized by the deposition in the myocardial interstitium of proteins with unstable tertiary structure that misfold, aggregate, and deposit as amyloid fibrils [6,55]. More than 30 proteins can misfold and deposit in tissues as amyloid fibrils, but almost all clinical cases of CA are from either misfolded monoclonal immunoglobulin light chains from an abnormal clonal proliferation of plasma cells (AL amyloidosis, also known as primary systemic) [56], or transthyretin (ATTR amyloidosis), a protein synthesized by the liver and normally involved in the transportation of the hormone thyroxine and retinol-binding protein [57]. Furthermore, ATTR may be either hereditary, arising from misfolded mutated TTR (ATTRm) [58], or nonhereditary, from misfolded wild-type TTR (ATTRwt, previously known as senile systemic amyloidosis) [59].

Considering the adverse prognosis of untreated cardiac AL amyloidosis [60], and the novel pharmaceutical strategies for cardiac ATTR amyloidosis [61], the diagnosis of cardiac involvement is of paramount importance. In addition to general clinical assessment with functional status evaluation and hematological assessment with serum free light chain assay along with serum and urine immunofixation electrophoresis in the setting of suspected AL amyloidosis, cardiac imaging is the key approach to detect myocardial amyloid infiltration. Echocardiography is the first-line imaging technique for patients presenting with suspected cardiac amyloidosis, but correct diagnosis is frequently delayed or missed, especially due to the limited sensitivity and specificity of the combination of wall thickness and diastolic function parameters such as transmitral flow deceleration time, E wave/A wave ratio and E wave/e’ wave ratio, with increased diagnostic performance if myocardial strain analysis is incorporated in a multiparametric evaluation [62]. Technetium-labeled bone scintigraphy allows to diagnose ATTR subtype with very high diagnostic accuracy, but it has no clinical role in the assessment of AL subtype [63]. Conversely, CMR with late gadolinium enhancement (LGE) assessment was shown to be the ideal imaging technique to detect cardiac involvement in both AL and ATTR subtypes [64,65] thanks to the typical diffuse subendocardial or transmural LGE pattern coupled with abnormal gadolinium kinetics, and also to add robust prognostication [66].

CA was one of the first myocardial disease assessed with mapping sequences in light of characteristic LV hypertrophy and diffuse cardiac muscle involvement. Karamitsos et al. tested the potential role of native T1 mapping by ShMOLLI sequence in 53 patients with AL amyloidosis, showing an area under the curve (AUC) for the detection of definite or possible cardiac involvement of 0.97 [67]. Fontana et al. tested the diagnostic performance of native T1 mapping also in ATTR, showing how T1 was elevated in 85 ATTR patients compared with hypertrophic cardiomyopathy and normal subjects, with a diagnostic accuracy comparable to AL amyloidosis, but with values not as high as the latter [68]. However, these studies were of retrospective nature, with small sample size, leaving a knowledge gap on clinical utility and accuracy of native T1 as a diagnostic test. Recently the diagnostic role of native T1 in a prospective cohort of 868 patients referred to a tertiary center for suspected systemic amyloidosis has been investigated, showing how native T1 was associated with high diagnostic accuracy in both AL and ATTR amyloidosis (AUC for overall population of 0.93), and suggesting cut-off values associated to optimal negative and positive predictive values, in order to exclude or confirm cardiac involvement with non-contrast CMR exams, and possibly to restrict the administration of contrast only to patients with intermediate probability (native T1 values between suggested cut-offs) [69]. Interestingly, it was shown how diagnostic accuracy of native T1 in detecting CA could be considered an early disease marker as it was not influenced by LV hypertrophy, and how it was not influenced by other relevant clinical manifestations, as renal involvement or anemia [69]. When the diagnostic accuracy of native T1 was compared against other CMR, clinical, and echocardiographic diagnostic markers, native T1 showed the best diagnostic accuracy with the only exception of ECV, a direct measure of the extracellular space, though linked to gadolinium-based contrast administration [69].

From a prognostic point of view, Banypersad et al. demonstrated that in 100 patients affected by AL amyloidosis, a disease often characterized by abrupt onset and extracellular amyloid deposition coupled with acute myocardial cells injury, both native T1 mapping and ECV are good predictors of mortality [70]. Conversely, in ATTR amyloidosis, Martinez-Naharro et al. showed that both native T1 and ECV correlates with mortality, but only ECV remains independently predictive of prognosis, underlining the superiority of ECV in this specific setting, being a better marker of amyloid deposition, the main driver of disease progression [71].

**Figure 3:**
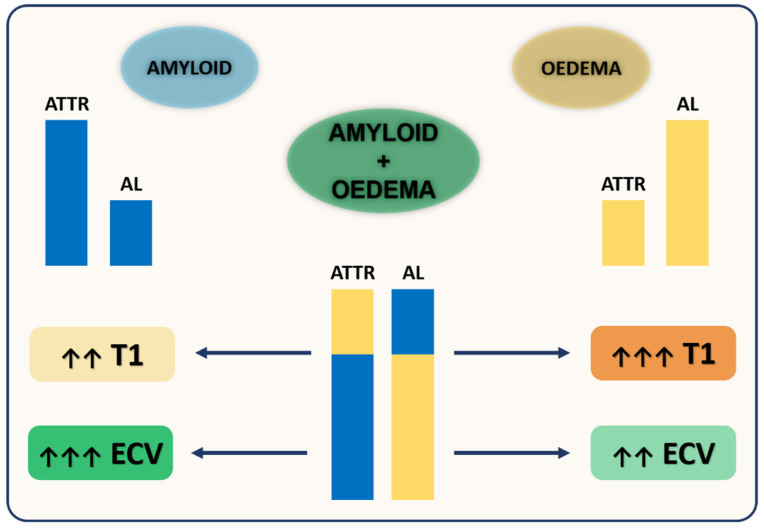
Different biological information provided by native T1 and ECV in Cardiac Amyloidosis (CA). Native myocardial T1 is highly influenced by water content in the tissue, thus significantly raised by oedema, at both intra- and extracellular level in amyloid light chains (AL) CA, or mostly at extracellular level in transthyretin (ATTR) CA. Conversely, ECV values are closely related to amyloid burden, and therefore are usually remarkably high in ATTR as compared to AL. This insight could explain the relatively higher T1 values in AL compared to ATTR, along with the higher ECV values in ATTR compared to AL.

These differences in diagnostic and prognostic performance of native T1 and ECV in AL and ATTR amyloidosis are believed to be related to different biological information provided by native T1 and ECV. Native T1 provides a composite signal from the intra- and extracellular spaces, thus potentially influenced by other pathophysiological mechanisms beyond amyloid burden, while ECV directly measures the extracellular space. Native myocardial T1 is highly influenced by water content in the tissue, and therefore, will be significantly raised by the presence of myocardial oedema, at both intra- and extracellular level, as occurs in AL due to light-chain toxicity and high rate of amyloid deposition, or mostly at extracellular level, as occurs in ATTR. Conversely, ECV values, being closely related to amyloid burden, are usually remarkably high in ATTR, where amyloid deposition is more abundant but usually more diluted over time as compared to AL. This insight could explain the relatively higher T1 values in AL compared to ATTR, along with the higher ECV values in ATTR compared to AL [70,71] (Figure 3).

The role of myocardial oedema in CA, especially in AL amyloidosis, is further highlighted by the study from Kotecha et al., in which the role and prognostic significance of myocardial edema and T2 mapping changes in patients with CA were investigated. In a cohort of 286 patients with AL amyloidosis, ATTR amyloidosis, suspected ATTR amyloidosis or asymptomatic individuals with amyloidogenic transthyretin gene mutations, T2 was increased in amyloidosis with the highest degree of elevation in untreated AL patients, followed by treated AL patients and then ATTR subjects. From a prognostic perspective, myocardial T2 sharply stratified prognosis in AL amyloidosis using a cut-off value of 55 ms, maintained prognostic power even if adjusted for other relevant parameters as ECV and N-terminal pro-hormone B-type natriuretic peptide (NT-proBNP), while did not predict mortality in ATTR amyloidosis [72].

Considering the remarkable evidence available, it is strongly suggested to assess every patient with suspected or known AL or ATTR amyloidosis with CMR and mapping tools, integrating such data with clinical, laboratoristic, echocardiographic, and nuclear imaging results.

**Figure 4:**
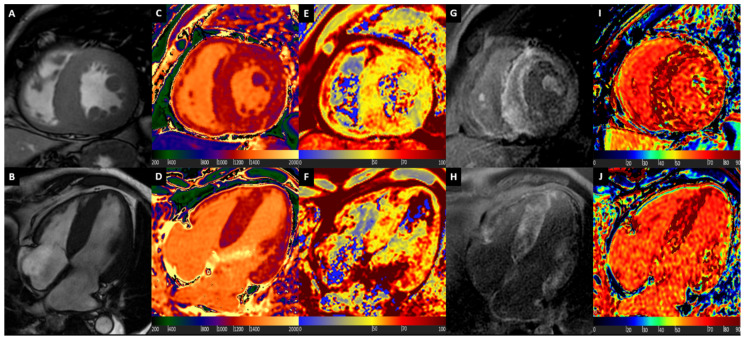
ATTR Cardiac Amyloidosis. Basal short axis view (Box **A**) and 4-chamber long axis view (Box **B**) balanced steady state free precession images showing diffuse myocardial hypertrophy and reduced longitudinal systolic function, mostly at basal and mid segments. Native T1 mapping shows diffuse marked increase of T1 values, with granular appearance (Boxes **C** and **D**). Myocardial oedema detected by T2 mapping (Boxes **E** and **F**), at basal segments. Late gadolinium enhancement (LGE) images show abnormal gadolinium kinetics, coupled with almost transmural enhancement at basal septum, and diffuse subendocardial enhancement at other segments (Boxes **G** and **H**). Diffuse severe ECV expansion is noted, up to more than 80% at septal level (Boxes **I** and **J**).

Figure 4 shows a comprehensive CMR assessment of a patient with ATTR Amyloidosis.

## 5. Cardiac Sarcoidosis

Sarcoidosis is a multisystem, granulomatous disease of unknown etiology [73], and strong evidence suggests that it is caused by an immunological response to an unidentified antigenic trigger, mostly in genetically susceptible persons. Noncaseating granulomas are the histopathological hallmark, typically affecting the lungs, as up to 90% of patients could present pulmonary involvement [74]; however, being multisystemic, also other organs as cardiac muscle, liver, spleen, eyes, parotid gland, and skin can be involved. Cardiac sarcoidosis (CS) represents one of the most relevant drivers of prognosis, considering the high rate of atrioventricular blocks, heart failure development and sudden cardiac death if heart is affected, as demonstrated by Ekstrӧm et al. in a large registry of 351 subjects with definite diagnosis of CS [75]. In light of such potential negative prognosis, timely diagnosis of myocardial involvement is of paramount clinical relevance, and thus an updated Expert Consensus Document was released in 2014 suggesting diagnostic algorithms in several scenarios in which a cardiac localization could be present [76]: according to clinical likelihood of CS (clinical history, EKG findings), CMR is suggested as first or second line test. Mostly, CMR is performed to obtain precise biventricular size, wall thickness and ejection fraction quantification, and to assess tissue characterization, usually with T2W and LGE images, because of its relevant diagnostic [77] and prognostic [78] role. Keeping in mind that there is no specific pattern of LGE that is typical for CS, usually the most commonly described LGE pattern is one or more patchy regions of myocardial enhancement that would be atypical for myocardial infarction (i.e., focal or diffuse subepicardial or mid-wall, and eventually focally transmural with or without typical granuloma) [79]; however, sometimes the correct identification of cardiac involvement remains challenging, as many other patterns of LGE and even a pattern in keeping for prior myocardial infarction could be present in CS [80].

The first relevant experience of application of mapping technique in this clinical context was performed by Greulich et al., who prospectively assessed 61 patients with systemic sarcoidosis and no symptoms or unspecific symptoms with a comprehensive approach composed by LGE-CMR, native T1 mapping, T2 mapping, and ECV, having 26 healthy volunteers as control group. Compared to healthy subjects, patients with systemic sarcoidosis showed higher T1, extracellular volume, and T2 values, with most significant differences for native T1 and T2 [81]. Interestingly, significant differences in T1, ECV, and T2 values compared with controls were detectable also in LGE-negative patients, suggesting a subclinical myocardial involvement of CS [81]. Moreover, in line with a retrospective analysis by Crouser et al., focused on T2 mapping of 50 subjects with histologically proven sarcoidosis, T2 mapping seemed to provide complementary information to detect CS beside the use of LGE-CMR, as sarcoid patients with ECG abnormalities showed significantly higher T2 values and a greater prevalence of LGE compared with patients with normal ECG, and 22% of the LGE-negative patients demonstrated T2 abnormality [82].

Puntmann et al. prospectively evaluated 53 consecutive patients with a biopsy-proven extracardiac diagnosis of systemic sarcoidosis and a control group of 36 volunteers with LGE-CMR, native T1 mapping and T2 mapping; furthermore, a follow-up study was performed in 40 sarcoid patients. In their work, Puntmann et al. demonstrated that native T1 mapping and T2 mapping were significantly higher in patients with sarcoidosis, irrespective of the presence of symptoms, age, or disease duration, and showed a linear correlation with LV mass [83]. Both native T1 and T2 had higher discriminatory accuracy when compared with traditional diagnostic criteria provided by Japanese Ministry of Health and Welfare and Heart Rhythm Society, with native T1 that resulted the strongest discriminator between health and disease. Finally, after a mean follow-up of 144 days, T1 and T2 mapping values had a significant reduction in the treated group but not in the untreated group, along with C-reactive protein levels, suggesting a potential role of CMR with mapping techniques in recognition of activity of myocardial inflammation and in tracking the response to anti-inflammatory treatment [83].

Considering the latter aim, the experience from Isted et al. is noteworthy. The Authors performed a CMR with native T1 mapping and LGE in a 56-year-old Caucasian female who developed heart failure symptoms over the preceding six months; in light of biventricular dilatation with moderate-severe systolic LV impairment, a thinned and aneurysmatic appearance at basal segments (anterior, anteroseptal, and inferior segments), extensive diffuse non-ischemic LGE (epicardial-intramyocardial and transmural distribution) in thinned basal segments and native T1 values raised at mildly thickened septum by more than five standard deviations (SD) above the reference range (suggesting active inflammatory process), suspicion of CS was raised. The diagnosis was confirmed after positive thoracic fluorodeoxyglucose (^18^F)PET scan, also positive at myocardial level, and biopsy of high (^18^F)PET-uptake thoracic lymph node. Accordingly, patient was treated with corticosteroid and optimized heart failure therapy, had a good symptomatic response, being symptom-free at regular follow-up appointments. After two years, as new onset of symptoms occurred, CMR was repeated, with stable functional parameters, unchanged distribution and extent of LGE, but with native T1 values at 2SD above the mean of the normal range, suggesting residual myocardial inflammation, and leading to another corticosteroid therapy cycle, that stabilized again the clinical status [84]. This case perfectly reflects the notion that tissue characterization with CMR and mapping techniques could offer, along with a more accurate detection of myocardial involvement, a more sensitive assessment of disease activity and response to anti-inflammatory therapy.

Finally, Nakamori et al. recently published a prospective study in 58 subjects with definite systemic CS undergoing LGE CMR evaluation for possible cardiac involvement, applying segmental analysis of native T1 and T2 mapping with five short axis slices, and having 58 age- and sex-matched control subjects. 11 patients were identified with CS, and at a median follow-up of 28 months, 6 possible CS patients without any LGE abnormalities on baseline CMR developed overt CS; in such patients, abnormal native T1 and T2 values were more prevalent in basal slice segments, with segmental but not global native T1 assessment discriminating among possible and confirmed CS, with a better diagnostic performance if compared to functional parameters such as global longitudinal strain (GLS) and LV ejection fraction (LVEF). This evidence suggests that changes in LV myocardial tissue occur before LGE abnormalities and subclinical LV dysfunction, highlighting the possible role of segmental mapping assessment for early detection of CS [85].

As previously mentioned, CMR is suggested as first or second line test in case of suspicion of CS. However, in light of high sensitivity in depicting cardiac involvement since early stages, timely performance of CMR scan with complete tissue characterization should be pursued as much as possible.

**Figure 5:**
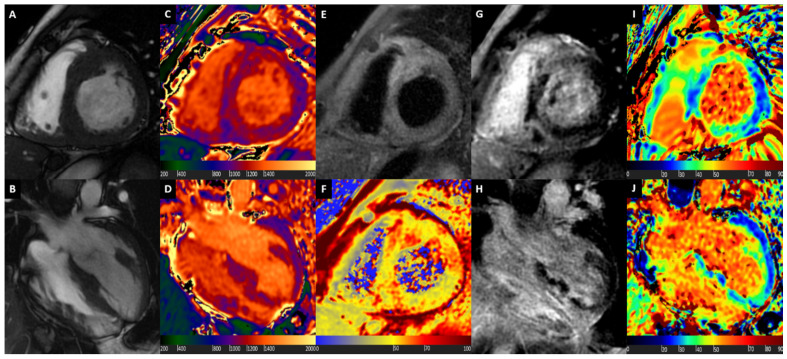
Cardiac Sarcoidosis. Basal short axis view (Box **A**) and 4-chamber long axis view (Box **B**) balanced steady state free precession images showing asymmetric myocardial hypertrophy at septum. Focal increase of T1 values at native T1 mapping images at septum and inferior wall (Boxes **C** and **D**). Myocardial oedema detected by T2-wieghted images (Box **E**) and T2 mapping (Box **F**), mostly at septum and inferior wall. LGE images show transmural enhancement at septal level, and subepicardial enhancement at inferior wall (Boxes **G** and **H**). Focal increase of ECV, matching native T1 mapping and LGE images abnormalities (Boxes **I** and **J**).

A representative case of patient diagnosed with CS with multiparametric mapping and LGE CMR is displayed in Figure 5.

## 6. Fabry Disease

Fabry disease (also known as Anderson–Fabry disease) is an X-linked inherited lysosomal storage disease caused by a mutation in the α-galactosidase A gene that results in lysosomal accumulation of globotriaosylceramide in different cells, including endothelial cells, cardiomyocytes, renal glomerular, and tubular cells, and in neurons in autonomic and dorsal root ganglia [86]. Fabry disease is rare, with an annual incidence of 1 in 100,000 [86], and clinical manifestations of the disease are diverse and nonspecific, making Fabry disease diagnosis challenging. In classic Fabry disease, affecting predominantly hemizygous males, cardiac involvement is present in 80% of patients, and cardiac manifestations occur later than neurologic, dermatologic, and renal manifestations, with a mean age of onset of 42. As the disease progresses, chronic kidney disease, cerebrovascular disease and cardiac involvement are the main determinants of morbidity and mortality. An atypical, late onset, predominantly cardiac variant has also been described, and it is thought to be responsible of up to 6% of cases of unexplained left ventricular hypertrophy (LVH) [87,88]. Initial work-up in males comprises measuring leukocyte α-galactosidase A activity; diagnosis is strongly suspected in males with a reduced leukocyte α-galactosidase A activity and confirmed by genetic testing. Genetic testing is required to establish the diagnosis of Fabry disease in females instead [89].

Physicians should suspect Fabry disease in patients aged ≥30 (males) or ≥40 (females) with unexplained LVH, particularly if there are extracardiac signs such as neuropathic pain, chronic kidney disease, stroke, transient ischemic attack, angiokeratoma, or characteristic corneal opacities [90]. In patients with Fabry disease with cardiac involvement, echocardiography usually shows concentric, non-obstructive LVH with wall thickness ≥13 mm [91]. Mitral annulus tissue Doppler velocity reduction and longitudinal strain reduction precede LVH development in Fabry disease patients [92,93], but are non-specific signs.

CMR, due to its intrinsic tissue characterization ability, has been proposed in differentiating Fabry disease from other causes of left ventricular hypertrophy/thickening, in detecting early cardiac changes in mutation carriers (particularly in females), in aiding the decision to initiate enzyme replacement therapy, in monitoring therapy effects [94], and in stratifying prognosis, as an increased left ventricular mass is associated with ventricular arrhythmias [95]. After contrast injection, LGE is usually found in a mid-wall distribution in the basal inferolateral wall [42,95], and can precede LVH in 38% of males and 17% of female carriers of the mutation commonly associated with the cardiac later onset variant [96].

Mapping techniques have shed more light on our understanding of cardiac involvement in Fabry disease. As a consequence of lipid storage, longitudinal relaxation time is markedly shortened, thus reflecting in characteristic low native T1 values [97]. While Sado et al. showed that typical diffuse low T1 values allow to fully differentiate Fabry disease from other causes of left ventricular hypertrophy/thickening such as hypertrophic cardiomyopathy, aortic stenosis and AL amyloidosis [98], Thompson et al. demonstrated that T1 mapping is the most sensitive and specific cardiovascular MRI tool in patients with Fabry disease irrespective of sex, LV morphology and function [99]. Furthermore, a very interesting consequence of the high sensitivity in detecting myocardial sphingolipid accumulation is the role of early marker disease, as shown by Pica et al. in a population of 63 Fabry subjects, in which native T1 values reduction precedes LVH in 40% of cases [100]. Unlike the behavior in other myocardial segments, native T1 values could present normal values (pseudo-normalization) or even increased values, with intramyocardial distribution, at the basal inferolateral wall, typical location of mid-wall LGE [98,101].

Nordin et al. demonstrated in a group of 47 Fabry patients that T2, along with T1 values, were increased in inferolateral segments with LGE, and all patients with LGE had also increased blood troponin levels. According to these hematological and CMR findings, authors have speculated that LGE in the basal inferolateral wall represents oedema rather than scar, suggesting to consider Fabry disease with LGE not just a storage disease but rather a chronic inflammatory cardiomyopathy [101], highlighting the unique role of mapping technique in characterizing types and phases of myocardial diseases.

Moreover, T1 and T2 values could be nicely used to track response to enzyme replacement therapy (ERT). In a group of 20 patients starting ERT, compared to treatment-naive controls, after one year of treatment not only a small reduction in wall thickness and myocardial mass was found, but also a reduction in T1 lowering was detected [102]. Similarly, also T2 values, along with wall thickness and myocardial mass, were found to be definitely lower in patients receiving ERT after 48 months of treatment [103].

Finally, considering that lipids storage occurs at lysosomal level, thus involving exclusively the intracellular space, the extracellular matrix is not affected, and ECV values are mostly in the range of normality, only presenting increased values in the segments with LGE [97].

According to the evidence available, early detection of cardiac involvement, mostly with diffusely reduced T1 values, and resulting early ERT administration, should be the goal of every physician involved in management of patients with suspected Fabry disease, and this aim is undeniably linked to routine implementation of CMR with detailed tissue characterization in this specific clinical scenario.

**Figure 6:**
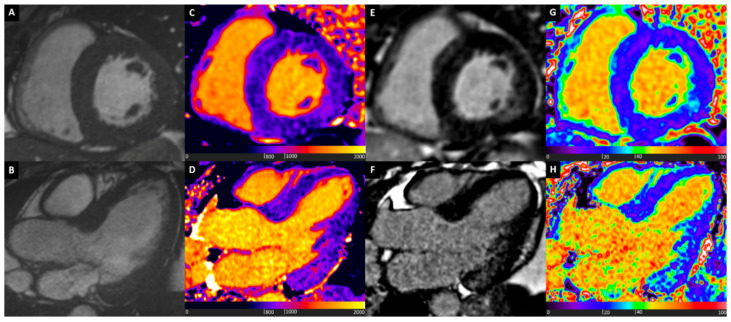
Fabry cardiomyopathy. Basal short axis (top row) and 3-chamber long axis (bottom row) cardiovascular magnetic resonance (CMR) images of a patient with Anderson–Fabry cardiomyopathy showing mild ventricular hypertrophy (Boxes **A** and **B**), low global native T1 values with normalized values at the basal inferolateral wall (Boxes **C** and **D**), mid-wall late gadolinium enhancement at the basal inferolateral wall (Boxes **E** and **F**), with matching high ECV values at basal inferolateral wall (Boxes **G** and **H**).

Typical CMR images of a patient with Fabry disease are shown in Figure 6.

## 7. Integration of Standard CMR Protocol with Mapping Techniques and LGE

In light of the evidences presented in this review, mapping techniques should be integrated in every CMR study performed in patient with suspected cardiomyopathy, especially if a hypertrophic phenotype is detected at morphological assessment. It is crucial to emphasize that informative data provided by mapping sequences have to be integrated to those provided by morphological analysis and, when possible, LGE assessment in order to correctly identify the underlying etiology and to prognosticate with the highest degree of confidence.

**Figure 7:**
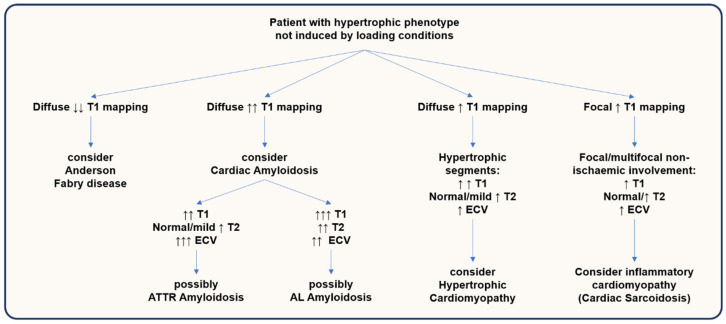
Etiological hypotheses of newly diagnosed myocardial hypertrophy according to mapping abnormalities. When informative data provided by mapping sequences are integrated to those delivered by clinical history, cardiovascular magnetic resonance (CMR) morphological analysis and, when possible, LGE assessment, differential diagnoses of Hypertrophic Cardiomyopathy, Cardiac Amyloidosis, Fabry disease or inflammatory cardiomyopathy such as Cardiac Sarcoidosis could be established with the highest degree of confidence.

Figure 7 summarizes different scenarios when a patient is found to have hypertrophic myocardial segments not induced by loading condition.

If concentric hypertrophy is found, in presence of diffuse reduction of T1 values, absence of LGE or small amount of LGE at basal inferolateral segments (and matching increase of ECV), diagnosis of Fabry disease should be suspected, with pseudo-normalization of T1 values and increase of T2 values at the level of the basal inferolateral segment suggesting an active stage of the disease.

If myocardial hypertrophy is detected, in presence of marked and diffuse increase of T1 values, coupled with abnormal gadolinium kinetics and peculiar LGE appearance, cardiac amyloidosis should be suspected. Furthermore, in presence of relatively higher increase of wall thickness and LV mass, and relatively higher increase of ECV compared to native T1, with normal or mildly elevated T2 values, ATTR Amyloidosis could be hypothesized; conversely, in presence of relatively higher increase of native T1 and T2 values compared to ECV, in the setting of relatively lower increase wall thickness and LV mass, AL Amyloidosis could be suspected. Anyway, technetium-labeled bone scintigraphy and full hematological assessment with serum free light chain assay along with serum and urine immunofixation electrophoresis have to be prescribed in order to complete the diagnostic evaluation.

If segmental hypertrophy is found, mostly at septal or apical level, with diffuse mild increase of native T1 values, and highest increase of T1 values at more hypertrophic segments, possibly coupled with matched typical LGE and increased ECV values, diagnosis of hypertrophic cardiomyopathy should be suggested.

Finally, if segmental myocardial hypertrophy is present, coupled with focal or multifocal increase with variable degree of native T1, T2, and ECV mapping, and matching LGE (usually with non-ischemic distribution but rarely with ischemic pattern), an inflammatory cardiomyopathy should be suspected, with cardiac sarcoidosis as one of the main differential diagnosis.

## 8. Conclusions

Mapping sequences represent a novel CMR technique that should be routinely implemented in the assessment of cardiomyopathies, especially if increase in myocardial mass is detected. As documented in this review, most of detailed tissue characterization is achievable avoiding contrast medium administration thanks to non-contrast T1 and T2 mapping. However, when feasible, contrast should be injected in order to add LGE imaging and ECV assessment, thus considering non-contrast mapping a complement rather than a substitute of traditional contrast-based tissue characterization. With this approach, CMR assessment of cardiac muscle disease is able to reach incomparable diagnostic and prognostic performance.
